# Median Age at HPV Infection Among Women in the United States: A Model-Based Analysis Informed by Real-world Data

**DOI:** 10.1093/ofid/ofab111

**Published:** 2021-03-12

**Authors:** Vimalanand S Prabhu, Craig S Roberts, Smita Kothari, Linda Niccolai

**Affiliations:** 1 Merck & Co., Inc., Kenilworth, New Jersey, USA; 2 Yale School of Public Health and Connecticut Emerging Infections Program, New Haven, Connecticut, USA

**Keywords:** cervical intraepithelial neoplasia, human papillomavirus (HPV), HPV acquisition, infection, median age at CIN2+ diagnosis, median age at HPV acquisition

## Abstract

**Background:**

The US Advisory Committee for Immunization Practices (ACIP) recommended shared clinical decision-making for human papillomavirus (HPV) vaccination of individuals aged 27 to 45 years (mid-adults) in June 2019. Determining the median age at causal HPV infection and CIN2+ diagnosis based on the natural history of HPV disease can help elucidate the incidence of HPV infections and the potential benefits of vaccination in mid-adults.

**Methods:**

Real-world data on CIN2+ diagnosis from the prevaccine era were sourced from a statewide surveillance registry in Connecticut. Age distribution of CIN2+ diagnosis in 2008 and 2009 was estimated. A discrete event simulation model was developed to predict the age distribution of causal HPV infection. The optimal age distribution of causal HPV infection provided the best goodness-of-fit statistic to compare the predicted vs real-world age distribution of CIN2+ diagnosis.

**Results:**

The median age at CIN2+ diagnosis from 2008 through 2009 in Connecticut was 28 years. The predicted median age at causal HPV infection was estimated to be 23.9 years. There was a difference of 5.2 years in the median age at acquisition of causal HPV infection and the median age at CIN2+ diagnosis.

**Conclusions:**

Real-world data on CIN2+ diagnosis and model-based analysis indicate a substantial burden of infection and disease among women aged 27 years or older, which supports the ACIP recommendation to vaccinate some mid-adults. When natural history is known, this novel approach can also help determine the timing of causal infections for other commonly asymptomatic infectious diseases.

Human papillomavirus (HPV) can cause cervical intraepithelial neoplasia (CIN), as well as cervical, vaginal, vulvar, anal, oropharyngeal, and penile cancers [1]. In the United States, there were more than 12 000 newly diagnosed cases of cervical cancer each year from 2012 to 2016, and over 4000 women died of the disease [2, 3]. HPV imposes a substantial burden among people aged 27 to 45 years, referred to as mid-adults [4]. Between 2008 and 2016, population-based data from 5 states from the HPV Impact Monitoring Project (HPV-IMPACT) revealed that while the incidence of cervical precancerous lesion diagnoses per 100 000 women declined for women aged 18 to 24 years, rates increased significantly for women aged 40 to 64 years [5]. The prevalence of penile high-risk HPV was highest among men aged 25 to 29 years (33.0%), with another peak at older age [6]. Oral high-risk HPV prevalence was highest among men and women aged 50–54 years [7]. Seroprevalence was highest among women aged 30–39 years and men aged 40–49 years [8], indicating a continued burden of HPV infection after age 27 years.

HPV infection and HPV-related diseases can be prevented by vaccinating with the nonvalent vaccine (9vHPV vaccine that targets HPV types 6/11/16/18/31/33/45/52/58) [9, 10]. The 9vHPV vaccine has demonstrated efficacy in preventing HPV infection and disease among mid-adults [11–13]. In 2018, through a priority review, the Food and Drug Administration (FDA) expanded the indicated age of the 9vHPV vaccine from a maximum of 26 years to 45 years [14]. Subsequently, in June 2019, the Advisory Committee on Immunization Practices (ACIP) recommended routine vaccination of girls and boys aged 11 to 12 years (vaccination can begin at age 9 years), catch-up vaccination for both women and men through 26 years, and shared clinical decision-making for mid-adults [15]. Health economic analysis from 5 different models of mid-adult vaccination were presented to the ACIP in 2019. The estimated economic benefits varied substantially for mid-adults [16–19], even when they estimated similar economic benefits for adolescent vaccination [20]. These are complex simulation models [18–22] that depend upon numerous assumptions regarding the natural history of HPV infection, sexual behavior, and HPV transmission. The differences in models may be difficult to resolve without a detailed cross-validation exercise. A simpler approach to understanding the potential value of mid-adult vaccination is needed. One such approach is to estimate the median age at causal HPV infection. A higher median age at infection would indicate that a substantial number of infections continue to occur among older individuals, which could justify vaccination of some mid-adults.

Determining the median age at causal HPV infection is challenging because HPV infection is often asymptomatic. There are aspects of HPV’s natural history that are “known” (can be observed) and “unknown.” The observable aspects include time from HPV infection to CIN2+ onset, the age distribution of CIN2+ diagnoses, and the screening patterns that result in CIN2+ diagnoses. Unknown data include the age distribution of causal HPV infection and the age distribution of CIN2+ onset. A simple approach could rely on “known” parameters from the peer-reviewed literature or use observable real-world data and then use a simple model to estimate only the unknown parameters. The median age at HPV infection has been estimated before using some of the complex models mentioned above [19, 21]. However, a simple approach to estimating median age at HPV infection has not been reported.

The objectives of this study are (1) to determine the median age at CIN2+ diagnosis using real-world data from the pre-vaccine era and (2) to predict the median age at causal HPV infection using a simple, model-based approach.

## METHODS

### Estimation of Age Distribution and Median Age at CIN2+ Diagnosis

We conducted a retrospective analysis of a statewide surveillance registry in Connecticut to estimate the age distribution of CIN2+ (CIN2, CIN2/3, CIN3, and adenocarcinoma in situ [AIS]) diagnosis. This registry covers the entire population of Connecticut from all 34 pathology laboratories that serve Connecticut residents. On January 1, 2008, the Connecticut Department of Public Health added CIN2+ to the state laboratory reportable disease list [23,24]. The reports contain information on pathological diagnoses and patient demographics. Data collected were subjected to quality assurance protocols, with duplicates removed from the data set. Only the first diagnosis of the highest-grade CIN2+ for each woman was included. This analysis was conducted in accordance with guidelines for good pharmacoepidemiology practices.

To assess the age of causal HPV infection in a way that meaningfully reflects future disease risk, we based our analysis on pre–vaccination era data. For this analysis, we used CIN2+ reported from January 1, 2008, through December 31, 2009. These were the earliest data available, and as vaccination was only introduced in late 2006, they corresponded closely to pre–vaccine era incidence. Women of all ages were included; no samples were excluded. Data on the number of diagnosed cases of CIN2+ by age were used to calculate descriptive statistics such as mean, median, and standard deviation (SD). A cumulative age distribution of CIN2+ diagnosis was plotted. The number of incident cases was divided by the target population to estimate the crude CIN2+ incidence (reported in the Supplementary Data).

### Patient Consent Statement

Institutional review board approval was not obtained because this study was an analysis of de-identified secondary data.

### Discrete Event Simulation Model to Predict Distribution of HPV Infection

#### Model Structure

**Figure 1.**
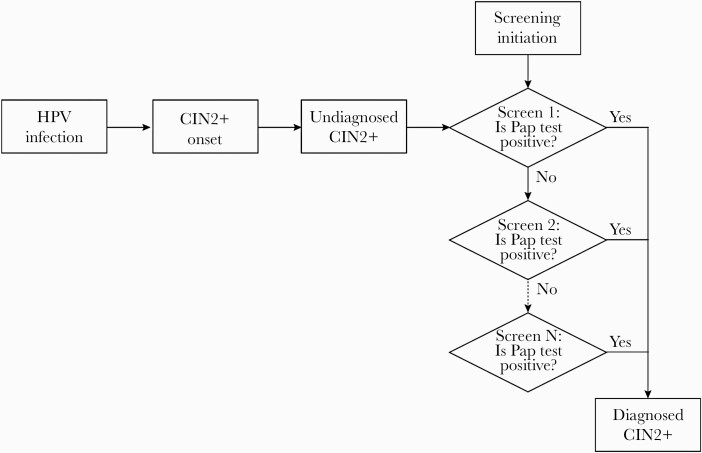
Discrete event simulation model structure. Abbreviation: HPV, human papillomavirus, CIN, cervical intraepithelial neoplasia.

We developed a simple discrete event simulation model to estimate the age of HPV infection as the difference between the age of diagnosis of CIN2+ and the time from HPV infection onset to CIN2+ onset. The model simulates age at causal HPV infection, age at CIN2+ onset, and age at diagnosis based on screening. The model had 3 health states: HPV infection, CIN2+ onset, and CIN2+ diagnosis following cervical screening (Figure 1).

The model simulates 1000 women who may be diagnosed with CIN2+ during their lifetime. Women enter the model at HPV infection onset or at age 18 years (start of screening), whichever occurs earlier. Age at HPV infection is determined endogenously within the model. We assumed that the unknown shape of the cumulative age distribution curve of causal HPV infection is similar to the cumulative age distribution curve of real-world data of CIN2+ diagnosis, except that it is shifted earlier by a fixed “offset.” This offset represents time from HPV infection to CIN2+ diagnosis.

Women with undiagnosed HPV infection can progress to CIN2+ onset (undiagnosed) based on a distribution for time from HPV infection to CIN2+ onset. Women with CIN2+ onset can then be diagnosed through screening. Time to next screening for women was estimated using an age-dependent exponential distribution that matched the proportion of women screened by age in a screening registry. Screening would result in diagnosis only if the woman had a CIN2+ onset and there was an accompanying positive test (determined by the Pap test’s sensitivity). Negative Pap tests resulted in women continuing to remain undiagnosed, undergoing subsequent screenings until diagnosed by a positive test, and then progressing to a diagnosed CIN2+ state.

We ran the simulation at different age-of-infection offsets from age of diagnosis at intervals of one-fifth of a year and then selected the offset that yielded the least chi-square goodness-of-fit statistic of comparison for predicted vs real-world Connecticut age distribution for CIN2+ diagnosis. The median for the age distribution at causal HPV infection associated with the optimal offset was determined to be the median age at HPV infection.

#### Model Inputs

We used data on time from HPV infection to CIN2+ onset from the VIVIANE bivalent HPV (2vHPV) vaccine clinical trials [25] and the FUTURE I quadrivalent HPV (4vHPV) vaccine clinical trials [26]. Data from the control arm of the 2vHPV trials showed that about 50% of infections progressed to CIN2+ within 1.5 years and 90% of infections cleared within 4 years (median, ~1 year). Of the HPV infections that progressed to CIN2 or CIN3 within 3 years in the placebo arms of the 4vHPV trials, 70%–100% progressed to CIN2 or CIN3 in 1 year (HPV types 16/31/45/52/58) and 73%–100% progressed to CIN2 or CIN3 in 2 years (HPV types 16/18/31/33/35/45/52/58) [26]. The median time from onset of persistent infection to CIN2+ was ≤1 year to 1.4 years in an internal analysis conducted on data from V503-001 (ages 16–26 years), V501-012 placebo (ages 16–23 years), and V501-019 placebo (ages 27–45 years) [27]. Based on this information, we assumed a Gamma distribution for time of HPV infection to CIN2+ onset (Gamma [α = 1, β = 1], with a 6-month offset, yielding a mean of 1.5 years and a median of 1.2 years).

For the base case, we assumed screening frequency from the New Mexico HPV Pap Registry (NMHPVPR) data from 2008 [28]. NMHPVPR is a population-based registry that monitors the full spectrum of cervical cancer preventive care. The percentage of the New Mexico population to receive a Pap test within the past year in 2008 was reported to be 22.4% for girls and women aged 15 to 20 years, 43.2% for women aged 21 through 29 years, 38.8% for women aged 30 through 39 years, 34% for women aged 40 through 49 years, and 28.2% for women aged 50 through 65 years. As our model assumed screening at age 18, we assumed the percentage of women screened from age 18 to 20 years to be 33.2%. An exponential distribution was used for time to screening, which was fitted to match the percentage screened per year by age from these data. The 2008 data were the most relevant, as these were closest to the pre-vaccine era and the year in which Connecticut real-world data were collected. We assumed the sensitivity of Pap tests to be 59% for the base case, with sensitivity analysis of 100% [29].

A sensitivity analysis was conducted using screening data from the Behavioral Risk Factor Surveillance System (BRFSS) survey conducted in Connecticut in 2008 [30] and varying the sensitivity of the Pap test from 59% to 100%. In this survey, the proportion of women who reported receiving a Pap test in the past 3 years was 91% among women aged 25 through 34 years, 91.6% among women aged 35 through 44 years, 90.6% among women aged 45 through 54 years, 86.5% among women aged 55 through 64 years, and 67.8% among women aged 65 years and older. As data from Connecticut were unavailable for women aged 18 through 24 years, we obtained data on this population from the neighboring state of New York. Incidences of AIS are rarer than CIN2/3 and could have a different median diagnosis age. Hence, a sensitivity analysis was conducted after excluding AIS.

## RESULTS

### Age Distribution of CIN2+ Diagnosis

**Figure 2.**
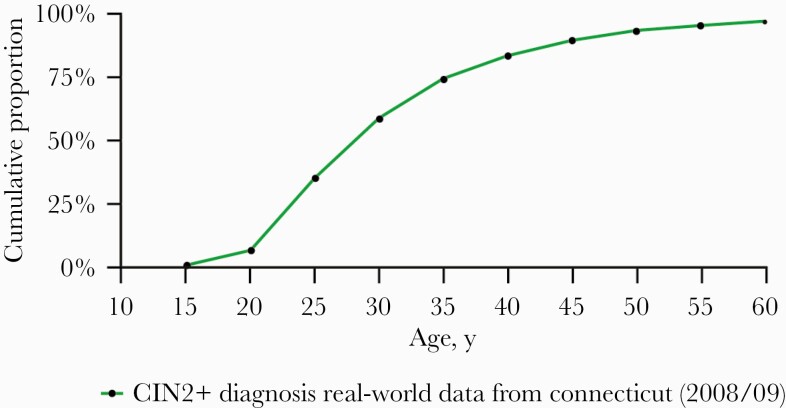
Cumulative distribution of CIN2+ diagnosis among women attending screening in Connecticut (2008–2009). Abbreviation: CIN, cervical intraepithelial neoplasia.

From 2008 through 2009, a total of 6083 cases of CIN2+ among unique female Connecticut residents were reported (Table 1). During this period, the median and mean ages of CIN2+ diagnosis were 28 and 31 years, respectively. The cumulative age distribution of CIN2+ diagnosis from 2008 through 2009 is reported in Figure 2. Most cases (3445) were CIN2. The median age at diagnosis increased with the severity of the high-grade lesions. The median age at CIN2 diagnosis (interquartile range [IQR]) was 26.0 (13.0) years in 2008 and 27.0 (12.0) years in 2009. The median age at CIN3 diagnosis (IQR) was 30.0 (19.0) years in 2008 and 31 (14.0) years in 2009. The median age at AIS diagnosis (IQR) was 37.5 (19.0) years in 2008 and 38.5 (10.0) years in 2009. Incidence rates are reported in the Supplementary Data.

**Table 1.** Annual Number of Cervical High-Grade Lesion Cases Among Females Screened For Cervical Disease, By Age and Diagnosis, 2008 and 2009 in Connecticut

	2008 (n = 3095)					2009 (n = 2988)				
Age group, y	AIS	CIN3	CIN2/3	CIN2	Total	AIS	CIN3	CIN2/3	CIN2	Total
0–14	0	0	0	1	1	0	1	0	1	2
15–19	0	34	31	134	199	0	14	21	92	127
20–24	6	180	131	551	868	6	143	108	604	861
25–29	7	200	133	348	688	2	156	110	428	696
30–34	6	140	79	224	449	9	140	81	233	463
35–39	10	98	54	123	285	7	88	34	149	278
40–44	8	66	34	116	224	13	63	37	112	225
45–49	4	42	26	86	158	5	51	24	69	149
50–54	3	26	10	44	83	4	7	13	45	69
55–59	1	18	17	19	55	0	17	10	26	53
60–64	3	19	12	7	41	0	15	7	13	35
65–69	1	12	3	5	21	0	6	5	5	16
70–74	0	9	1	3	13	0	4	1	1	6
75–79	1	3	0	3	7	0	3	0	0	3
80+	0	3	0	0	3	0	1	1	3	5
Total	50	850	531	1664	3095	46	709	452	1781	2988
Mean age of diagnosis ± SD, y	39.0 ± 12.7	33.2 ± 12.2	31.4 ± 10.9	29.5 ± 10.0	30.97 ± 11.0	37.1 ± 8.7	33.5 ± 11.3	31.9 ± 11.2	29.6 ± 9.8	31.0 ± 10.5
Median age at diagnosis/IQR, y	37.5/19.0	30.0/14.0	28.0/14.0	26.0/12.0	28.0/13.0	38.5/10.0	31.0/14.0	29.0/13.0	27.0/12.0	28.0/13.0

Data comprise cases recorded at all 34 pathology laboratories that serve Connecticut residents.

Abbreviations: AIS, adenocarcinoma in situ; CIN, cervical intraepithelial neoplasia; IQR, interquartile range.

### Median Age at Causal HPV Infection

**Figure 3.**
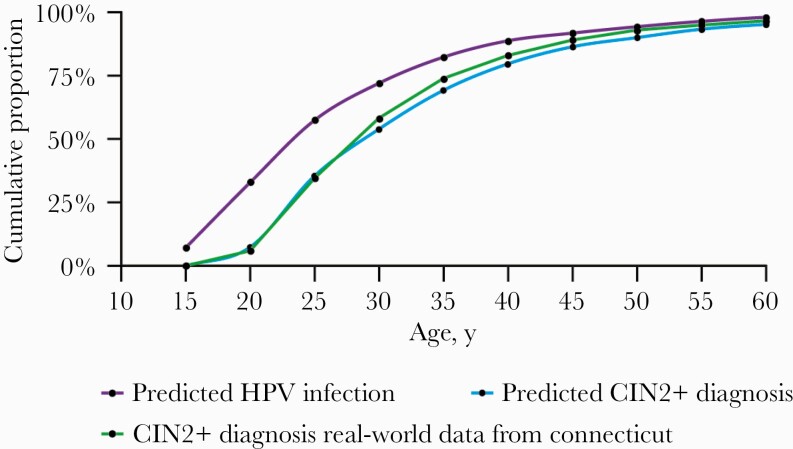
Cumulative distribution of predicted age distribution of human papillomavirus infection. Abbreviation: HPV, human papillomavirus.

Figure 3 shows the cumulative age distribution for predicted HPV infection, predicted CIN2+ diagnosis, and the real-world data on CIN2+. The optimal mean time between predicted HPV infection and observed CIN2+ diagnosis for the base case was 5.2 years (Table 2). The estimated median age at causal HPV infection was 23.9 years. Approximately 42.7% of causal infections among women occurred at age 27 years or older. The median age was 24.7 years with BRFSS screening data and 59% sensitivity, and 25.6 years when we assumed 100% sensitivity of the Pap test, irrespective of the underlying screening algorithm. After excluding AIS from the analysis, the median and IQR for age at CIN2+ diagnosis did not change; the optimal mean time between predicted HPV infection and observed CIN2+ diagnosis and median age at causal infection remained almost the same, at 5.0 years and 24 years, respectively (Table 3).

**Table 2.** Optimal Offset and Median Age at Causal HPV Infection

Scenario	Optimal Offset Between Predicted HPV Infection Curve and Predicted CIN2+ Diagnosis Curve, y	Median Age at Causal HPV Infection, y
NMHPVPR screening, 59% sensitivity	5.2	23.9
NMHPVPR screening, 100% sensitivity	3.6	25.5
BRFSS screening, 59% sensitivity	4.4	24.7
BRFSS screening, 100% sensitivity	3.5	25.6

Abbreviations: BRFSS, Behavioral Risk Factor Surveillance System; CIN, cervical intraepithelial neoplasia; HPV, human papillomavirus; NMHPVPR, New Mexico HPV Pap Registry.

**Table 3.** Sensitivity Analysis After Excluding AIS

Estimate	Value
Median age at diagnosis for entire sample in 2008 after excluding AIS/IQR, y	28.0/13.0
Median age at diagnosis for entire sample i n 2009 after excluding AIS/IQR, y	28.0/13.0
Median age at casual HPV infection, y	24.0

Abbreviations: AIS, adenocarcinoma in situ; IQR, interquartile range.

## DISCUSSION

We developed a novel approach to use the current knowledge of natural history of HPV infection and real-world data on diagnosis of high-grade cervical lesions in a simple model to estimate the median age at causal HPV infection. Our findings indicate that the median age at causal HPV infection resulting in CIN2+ may be higher than an earlier model-based estimate by Burger et al. (2019), who estimated that when using perfect screening, more than half of cervical HPV infections that progress to high-grade cervical disease are acquired by the age of 21 years [21]. Our estimate of median age at HPV infection is consistent with a more recent estimate by Burger et al. of 25.1, 25.4, 27.9, and 49.9 years for the UMN-HPV CA, Harvard, Policy 1-Cervix, and MISCAN-Cervix models, respectively, when they assumed imperfect compliance (more representative with real world) with the US screening guidelines [19]. Our estimate is also consistent with Daniels et al. (2020) [18], who reported the median age at HPV infection to be 25 to 26 years. Our results show that half of the infections that may cause cancer occur after age 23.9 years, implying continued infection among mid-adults aged 27 to 45 years. This supports the ACIP guidelines for shared clinical decision-making among mid-adults [15]. Determining the median age at infection in the prevaccine era is important, as median age at infection is impacted by protection from vaccination in the postvaccination era and no longer allows for an insight into the proportion of disease that may be prevented in populations at different ages.

Although our approach was completely different, our results are similar to 3 of the 4 scenarios in Burger et al. (2019) [19] as well as those reported by Daniels et al. in 2020 [18]. Our model focused on causal infections that resulted in CIN2+ incidence in the prevaccine era under real-world screening in order to estimate the approximate age at infection. We chose this approach because there is less uncertainty in the natural history leading to CIN2+ regarding time of progression and because CIN2+ is a common manifestation of HPV-associated cervical disease in a highly screened population, such as that of the United States. We believe this approach provides a broad and generalizable estimate of the timing of causal HPV infections that result in disease. The age distribution along the predicted curve of age at infection can be useful to estimate the proportion of preventable diseases that may be achieved through vaccination of older female cohorts.

While our approach is also based on assumptions, similar to dynamic transmission models, our analysis requires only a minimal sequence of necessary data elements to reach its conclusion, each with relatively well-defined characterization of timing, specifically: age distribution of CIN2+ that is observable in the real world, screening frequencies and intervals observable in the population, and time from infection to disease as determined by natural history data from clinical trials. Dynamic transmission models are extremely complex and may use 100+ variables, with several hidden endogenous transitions, in order to answer a wide variety of policy questions. The simplicity of our model provides a direct and intuitive connection between the time from infection to CIN2+ onset and from CIN2+ onset to CIN2+ diagnosis. Our approach produces an answer consistent with the dynamic transmission model, adds to the range of possible estimates of the body of evidence on median age at HPV infection, and contributes to the literature by adding to the range of possible estimates for median age at causal infection.

The estimated median age at CIN2+ diagnosis in Connecticut at 28 years is consistent with several previously reported estimates in the United States. In 2008, the median age at CIN2+ diagnosis in the population-based HPV-IMPACT project conducted in sites in Oregon, California, New York, Connecticut, and Tennessee was 28 years [5]. Only 1 county in Connecticut (New Haven) reported data to the HPV-IMPACT project, while our study includes data from the whole state. Watson et al. reported a median age of 30 years at CIN3+ diagnosis based on data from central cancer registries, which included 2009–2012 data from Louisiana, Kentucky, and Missouri as well as 2011–2012 data from Los Angeles, California [31]. Data from 5 out of 7 large clinical centers in the United States from 2007 that were investigated by Castle et al. (2009) show a median age of 24 to 34 years at CIN2+ diagnosis [32].

We estimated a relatively brief period from HPV infection to CIN2+ diagnosis. Data on the natural history of HPV from clinical trials consistently support progression time from HPV infection to CIN2+ onset of between 1 and 2 years. Clinical trial data are ideal to study natural history because they use sensitive assays that allow for the adequate detection of HPV DNA and histologic end points that are HPV-typed and rigorously analyzed by a panel of pathologists. Trials include frequent data collection and follow-up conducted over a period of 4 to 5 years. A review and meta-analysis of studies published through January 1, 2009, reported an average median duration of cervical high-risk HPV infection of 9.3 months, which ranged between 6.0 and 14.8 months among the 15 studies analyzed. The weighted median duration of HPV persistence was 9.8 months (some included prevalent infection) [33].

A key parameter of interest in determining the median age at infection is the time from CIN2+ onset to CIN2+ diagnosis; in our study, it was around 3.6 to 4.1 years. Screening data from NMHPVPR [28] show that in 2008 the median time to next screening was 1.5 years for women aged 21 to 65 years. Assuming a sensitivity of about 59%, which might add another 2 years or so to diagnosis, a time lag of ~3.6 to 4.1 years between CIN2+ onset and CIN2+ diagnosis is intuitively reasonable. Results were not sensitive to exclusion of AIS from the analysis. This could be because AIS accounted for only 1.5% of the overall sample, and our primary analysis was focused on the median estimates.

Studies investigating mid-adult women have reported associations between HPV infection and risk behaviors, including recent new partners [34–36] and lifetime number of partners [35, 36]. Mid-adults can be exposed to HPV; in a 2007–2010 NHANES survey, 20% of women aged 18 to 45 years reported multiple sexual partners. The median number of lifetime partners increased with age, indicating acquisition of new partners through mid-adulthood [37]. About 70% of women under age 45 years at baseline in the quadrivalent vaccine trials (Protocol V501-013/15 and V501-019) had no anogenital HPV infection, and no women tested positive for all 9 types [27]. If exposed to HPV, they could get infected. Studies have shown that mid-adult persons can acquire new HPV infections [38–40]. Data from bivalent vaccine clinical trials have shown that HPV infections among women aged ≥25 years (VIVIANE study) progressed to high-grade disease at a similar rate to adolescents and women aged 15 to 25 years (PATRICIA study) [25].

Our model does not support the presumed traditional view that most HPV infections occur within 5 years of sexual debut [32]. Our model predicts that 42% of the causal infections occur among mid- and older adults; therefore, individual protection through HPV vaccination should be considered an important policy option to reduce the burden of HPV among mid-adults, as HPV vaccination efficacy has been demonstrated among women aged 24–45 years [11, 12]. We used pre–vaccine era data to model the median age at infection to ensure that we captured the epidemiologic pattern and natural history before any possible vaccine impact on these parameters. In recent years, the incidence of CIN2+ among younger women declined, and the median age at CIN2+ diagnosis increased from 28 years in 2008 and 32 years in 2016 [5]. These data also support the idea that adults continue to be infected and vaccination may still provide protection to some mid-adults who have not been vaccinated. While this novel analysis on the distribution of age of causal HPV infection can help inform the potential for HPV prevention for different age groups, it is one of a number of considerations. Other important considerations include immunogenicity and effectiveness of vaccination in older age groups, which is out of the scope of this analysis. In addition, it is also necessary to ensure compliance with screening guidelines and completion of the full recommended course of HPV vaccination, as the long-term durability of vaccination beyond the teenage and young adult years is necessary.

The simple approach used in the present study has the advantage of being readily understood and applied. Analysis on age at CIN2+ diagnosis was based on real-world patient outcomes data from pathology laboratories serving the whole state of Connecticut. Our study uses natural history from clinical trials and a high-quality CIN2+ registry to model screening modalities. However, there are a few potential limitations. Current knowledge regarding the natural history of HPV infection is incomplete. In particular, estimating the median age at women acquiring causal HPV infections can be complicated by the fact that cervical screening does not differentiate between lesions that result from newly acquired infections, those due to re-infection from recent sexual exposure, and those that result from HPV infections re-activating after a period of latency [41–43]. Connecticut data may not be generalizable to other states in the United States, as Connecticut has lower rates of poverty, smaller proportions of residents who are members of ethnic minorities, fewer urban areas, and lower incidence of cervical cancer than the rest of the United States. Data on cervical screening participation in New Mexico may not be representative of Connecticut or the rest of the United States. However, these data provide more conservative estimates than those from the BRFSS survey conducted in Connecticut. Our model does not include different oncologic HPV types because the underlying CIN2+ data from Connecticut were not typed. Published placebo data from 4VHPV trials [26] and our internal analysis [27] show that among those who progress the median time of progression was <1 year to 1.4 years, and our overall assumption regarding time from infection to CIN2+ onset is reasonable.

Our approach can be applied to other infectious diseases that are often asymptomatic. If the sequalae of an infection and its age distribution can be observed and the natural history of disease is well studied, this method can be used to estimate the age distribution of causal infection. The method is intuitive, easy to implement, and has limited data needs.

In summary, we provide evidence of a substantial burden of causal HPV infection and high-grade cervical disease among mid-adult women. We found that the interval between acquisition of HPV infection and diagnosis of CIN2+ was relatively short and resulted in an estimated median age at causal infection of 23.9 years. Our approach provides decision-makers with an alternative way to assess the potential benefit of HPV vaccination of mid-adult women and supports the current ACIP guidelines for vaccination of some mid-adults aged 27 through 45 years through shared clinical decision-making.

## Supplementary Material

ofab111_suppl_Supplementary_Data
